# A life uncertain – My baby’s vulnerability: Mothers’ lived experience of connection with their preterm infants in a Botswana neonatal intensive care unit

**DOI:** 10.4102/curationis.v39i1.1575

**Published:** 2016-08-30

**Authors:** Rosinah K. Ncube, Hilary Barlow, Pat M. Mayers

**Affiliations:** 1 Division of Nursing and Midwifery, University of Cape Town, South Africa; 2 Midwifery at Bamalete Lutheran School of Nursing, Oodi, Botswana; 3 School of Child and Adolescent Health, University of Cape Town, South Africa

## Abstract

**Background:**

Preterm and low–birth weight infants are often separated from their mothers when admitted to neonatal units for stabilisation of body temperature and technological support.

**Objectives:**

The aim of the study was to explore and describe the lived experiences of mothers regarding care of their hospitalised preterm infants in a neonatal unit in a public hospital in Gaborone, Botswana.

**Method:**

This study utilised a qualitative exploratory and descriptive phenomenological study design. Mothers of hospitalised preterm infants were purposefully selected, with whom there was extensive engagement. Two in-depth interviews were conducted with each participant (P).

**Results:**

Mothers were shocked by the sudden birth of a preterm infant and found the neonatal environment intimidating. This increased their fear and anxiety and delayed development of a relationship with their infants. Support from staff, other mothers in the neonatal unit and family members enabled the mothers to overcome their fear and to develop an emotional connection with their infants.

**Conclusion:**

On-going supportive communication with the mothers by healthcare professionals promotes their confidence and competence in caring for their preterm infants, which in turn promotes mother–infant attachment.

## Introduction and background

Internationally, neonatal deaths (an infant under 28 days of age) (US National Library of Medicine 2013) account for nearly 41% of all deaths among children under the age of 5 years. The majority of these deaths are in developing countries, and in particular in the African region (Oestergaard *et al*. 2011). In Botswana, the neonatal mortality rate in 2012 was 29 per 1000 live births (Countdown to 2015. Maternal, Newborn & Child Survival 2015). The global prevalence of low–birth weight (LBW) infants is 15.5%, and this contributes to 60–80% of all neonatal deaths (WHO 2016). LBW has been defined as weight less than 2500 g (WHO 2011a:1). A preterm or premature infant is one born at less than 259 days of gestation (Valero de Bernabe *et al*. 2004).

Africa and South Asia are the regions where the least progress in reducing neonatal deaths has been achieved over the period 2001–2011 (WHO 2011b). HIV in pregnancy contributes to LBW among neonates, with an associated high incidence of neonatal deaths (Chen *et al*. 2012; Creek *et al*. 2010). This is a complicating factor in Botswana, with its high prevalence of HIV among pregnant women (27.7% in 2010) (Unite for Children, Botswana 2012).

Specialised care is required for neonates in neonatal intensive care units (NICUs); the requirements for effective treatment of such neonates could overwhelm the healthcare system, especially in developing countries where there is a lack of equipment and shortage of adequately qualified healthcare personnel (Ruiz-Peláez, Charpak & Cuervo 2004).

LBW and preterm infants are unable to regulate their body temperature and are vulnerable to numerous complications, including respiratory problems, neonatal jaundice, feeding problems and neonatal infections (Eichenwald & Stark 2008; Lau & Morse 2003). Such infants require technological intervention and support, are nursed in NICUs (Cinar & Filiz 2006) and are separated from their mothers (Tilokskulchai *et al*. 2002).

A mother, unprepared for the birth of a preterm infant (Aagaard & Hall 2008), might experience difficulty in bonding with her vulnerable infant if separated from him or her because of admission to the NICU (Tilokskulchai *et al*. 2002). Mothers of LBW and preterm infants experience a higher incidence of psychological distress during the neonatal period (Holditch-Davis *et al*. 2003; Malakouti *et al*. 2013; Tilokskulchai *et al*. 2002). Maternal behaviour noted in these studies included fear of touching or harming their infants, emotional ambivalence and lack of confidence in their role as parents of a small and vulnerable infant, which is mitigated by the involvement of mothers in the care of their infants while in the NICU (Erlandsson & Fagerberg 2005). Maternal psychological distress has been reported to increase the child’s risk of developmental problems (Kingston 2011). Globally, initiatives aimed at minimising separation of mother and infant, promoting infant well-being and maternal coping skills include kangaroo mother care (Nyqvist *et al*. 2010), the Baby-Friendly Hospital Initiative (Nyqvist *et al*. 2013; Nyqvist & Kylberg 2008) and the Humane Neonatal Care Initiative (Charpak, Ruiz & Calume 2000).

### Problem statement

Despite increasing evidence that minimising separation from mother and infant soon after birth is beneficial for both, there remains some reluctance to implement this practice with neonates and preterm infants in developing countries (Charpak & Ruiz-Peláez 2006). Family-centred care in NICUs has not been optimally promoted (Obeidat, Bond & Clark 2009). Reasons for this may include health professionals’ lack of knowledge and expertise, as well as human and structural resource constraints (Hurst 2006; Irlam & Bruce 2002).

In Botswana, preterm infants are admitted to the health facility’s NICUs and the mothers are admitted to the postnatal ward. In the Setswana culture, the mother is the primary caregiver for an infant and no other relative is allowed access to the NICU (Datta 2007; Sedimo, Mbongwe & Kote 2010). Mothers have regulated and structured access to their infants in order to feed and interact with them. The Botswana Ministry of Health (MoH 2011) reported that none of the 34 hospitals assessed in Botswana in 2010 met the criteria to be declared as ‘baby-friendly’ based on global and national criteria. The criteria for a hospital’s baby-friendly accreditation include a written breastfeeding policy, appropriate training of healthcare staff for promotion of breastfeeding, rooming-in and support of breastfeeding mothers (UNICEF 1991).

### Aim of the study

The aim of the study was to explore and describe the lived experience of mothers with regard to the care of their preterm infants, within a hospital NICU environment in which mothers had restricted interaction with their preterm infants.

## Research method and design

The study was qualitative, explorative and contextual in nature, using Husserl’s descriptive phenomenology as the philosophical underpinning for the study (Lopez & Willis 2004). Descriptive phenomenology requires careful description of the individual’s experience and meaning attributed to the experience and that the phenomenon is explored ‘through direct interaction between the researcher and the objects of study’ (Kumar 2012:793). The phenomenon was the experience of mothers with preterm infants in the NICU.

### Setting

The study setting was a high-care infant unit/NICU in a referral hospital in Gaborone, Botswana, one of two referral hospitals in the country and the largest neonatal setting in the southern part of the country. This 40-bed unit, with intensive care beds for critically ill infants, admits preterm infants from the city as well from clinics and district hospitals in the south. There are approximately 1400 neonatal admissions per year (Mudzikati & Dramowski 2015).

### Study population and sample

Women whose preterm infants had been admitted to the hospital NICU immediately post-delivery were purposively sampled. Women who met the following inclusion criteria were invited to participate in the study: a woman who had delivered a singleton infant before 37 weeks of gestation (definition of preterm infant described previously and the assumption that the mother’s experience would be different for multiple preterm infants), whose infant had been hospitalised for more than 5 days (assuming that the mother had sufficient ‘experience‘ of the NICU to enable her to reflect on her experiences), stable or improved health of infant and able to communicate in Setswana or English. Women who had delivered a preterm infant who was acutely ill at the time of data collection or whose infant had a congenital abnormality were excluded from the study.

### Data collection

The study was conducted over the period December 2010 to January 2011. Eight mothers participated in the study. Participants’ ages ranged from 23 to 30 years. All eight infants had been admitted to the NICU for prematurity and its associated complications. One participant’s infant (P8) was transferred to the premature infants’ section after stabilisation, and one participant’s infant died from respiratory complications (P3); however, she requested to remain in the study. All preterm infants were nursed in incubators or radiant warmers (Ogunlesi 2009) in the premature infant section while receiving intravenous and oxygen therapy, until their condition had stabilised or they weighed 1300 g. Thereafter, they were nursed in cribs within the NICU until they had gained sufficient weight and were feeding satisfactorily. Infants are routinely discharged at 1800 g weight, if well and feeding has been established.

Two in-depth interviews with each participant were conducted in a quiet room in the postnatal ward of the study facility. The primary question asked of each participant was: ‘Tell me about your experience of having your baby cared for in the neonatal unit’. The first interview was conducted 5 days after admission of the preterm infant, and the second interview just prior to discharge of the infant. All interviews were conducted in Setswana and audio-recorded. In order to facilitate engagement with the participants, the first author, a nurse-midwife and Setswana speaker, spent time each day with the participants during the study data collection phase, especially when the mother was feeding her infant. Field notes were kept and used to inform the data analysis and facilitate the rich description of the lived experience. Data saturation was achieved after these participants had been interviewed, at which point no new information emerged and no further recruitment was done.

The primary research question ‘Tell me about your experience of having your infant cared for in the neonatal intensive care unit’, was followed, when necessary with prompt questions and reflections:

- Can you describe your feelings when you first saw your son/daughter?
- Since you first held him/her, how have your feelings changed?
- Describe anything in the neonatal intensive care unit that strongly affected you, for example: made you feel happy or sad, frightened or reassured?
- Tell me about your interaction with the medical/nursing staff in the neonatal intensive care unit.
- If there was anything that could have made this time easier for you and your family, what is it ?

### Data management and analysis

Interviews were transcribed verbatim in Setswana and then translated into English and checked for accuracy by a colleague fluent in both languages (Davidson 2009). Field notes were used to capture the pauses, laughter, tears, distractions and non-verbal communication during an interview (Bailey 2008; Hycner 1985). The transcripts were analysed using the approaches of Colaizzi (1978) and Hycner (1985). The transcripts were read and reread while listening to the audio-recordings. For each transcript, significant statements were extracted, initial meanings were formulated and the transcript reread to ensure fidelity to the participant’s words. This was repeated for each transcript. Meanings were then organised into clusters and checked against the data to validate the emerging patterns. Each cluster of themes was then examined. Five central themes emerged.

## Ethical considerations

Ethical approval was obtained from Human Research Ethics Committee, Faculty of Health Sciences of the University of Cape Town (HREC REF: 332/2010), the Ministry of Health of Botswana Health Research and Development Unit and the hospital Institutional Research and Ethics Committee. The study was conducted in accordance with the Declaration of Helsinki (World Medical Association 2008).

Permission for the study was obtained from the Hospital Superintendent, the Principal Nursing Officer and Head Nurse of the NICU. Mediated access to potential participants was negotiated through the duty nurse who informed the mothers in the unit about the study. Interested potential participants were provided with information about the study. Once participation was agreed to, formal written informed consent was obtained.

Participants were informed about the study purpose, confidentiality and the right to withdraw from the study without any penalty or risk to the care of their infants (Morse 2007). They were also informed that the study would not benefit them directly but that the information obtained through the study may influence nursing practice, therefore benefiting mothers and preterm infants in future. All transcripts were coded to protect identity and data safely stored.

Arrangements were made with hospital social workers for psychosocial support should any of the participants experience any emotional distress as a result of their participation. A particular ethical concern arose for the researchers, as a mother, who had agreed to participate, lost her infant shortly after the first interview. This participant was offered the opportunity to withdraw from the study; however, she requested to be allowed to reflect on the loss of her infant and her interview data were therefore included in the analysis. This highlights the importance of facilitating a trusting relationship with study participants so that their choice is respected, even in situations of emotional distress.

## Trustworthiness

The principles for ensuring trustworthiness were adhered to in the study. Prolonged engagement in the setting, accurate descriptions of the context, methodology, careful transcriptions of interviews and rich description of the participants’ lived experience all contribute to the credibility of qualitative research and have been applied in this study.

## Results

Five themes emerged which reflect the experience of mothers whose preterm infants were admitted to the NICU:

- A life uncertain – my baby’s vulnerability
- An unfamiliar and intimidating environment
- Interacting with health professionals
- From fear to emotional connections
- An enabling support network.

## A life uncertain – my baby’s vulnerability

The unexpected birth of a preterm infant was traumatic. Denied the psychological preparation for the birth during the third trimester, mothers experienced uncertainty, emotional and psychological stress and feared for the survival of their newborn infant. For this mother, her concern when realising her infant’s different appearance and vulnerability was evident:

‘After delivery … after delivering him … I did not expect that he will be so small. I was scared because I was even feeling cold and took off the clothes that I was wearing’ (P7).

It was difficult for the participants to take on their anticipated maternal caring roles. They yearned to handle their infants but felt incapable and afraid of doing so, fearing that handling could damage the baby. ‘Aah I was afraid of him; he was too small. I didn’t know how to handle him though I ended up handling him and changing his nappy’ (P1). The presumed vulnerability of the infant initially affected the interaction of the mother with the baby:

‘At first I was scared, seeing him … wondering how … am I going to handle such a very small baby? I also thought that … he was … very small and soft, handling him might hurt him since his hands are not yet proper.’ (P1)

It was difficult to believe that this tiny, frail and vulnerable infant would survive, and this made it even more difficult to invest in the new relationship:

‘The baby was too small … you could not believe that he will survive for a longer time. I used to see babies but I never saw a baby born that small. I was wondering … whether he will survive or not.’ (P2)

Fear and anxiety was at its greatest when an unexpected complication was observed, such as cessation of respiration: ‘I was frightened because he was not breathing. I was wondering whether he was dead or what was happening to him’ (P4). This participant described her immense fear when she found her infant with secretions from mouth and nostrils, as she thought he was not breathing: ‘He had a lot of foam from his mouth and yellow stuff from his nostrils … that frightened me as I was wondering … I thought he is not breathing well, you see…’ (P7).

Any perceived abnormality increased participants’ concern about their infants’ well-being and they needed explanations: ‘Last time I asked the nurse about the fact that my baby experienced eye discharges every time the light was put on and whether that does not affect him’ (P7).

The limited, although regular, 3-hourly contact with their infants was stressful, particularly as mothers became aware that each nurse cared for more than one infant. They wanted to be allowed to stay in the NICU to ensure the infant’s safety:

‘Some babies lose their lives because they were not identified or seen. So when you are there … going to the neonatal unit more often, I think it is safe …this would even assist nurses because I think some things happen when they are not aware….’ (P7)

## An unfamiliar and intimidating environment

The unfamiliar, intimidating environment of the neonatal unit exacerbated mothers’ anxieties. The equipment impeded easy contact, which was compounded by their awareness of many other sick infants:

‘You might have seen your baby but seeing so many of them is scary because some of them have many drips with so many other things on their chest and nostrils.’ (P7)

Participants wanted to be informed about the equipment and its function, and incubator alarms added to the anxiety:

‘You know where those babies are … when you get there, you do not know the equipment … the first thing that frightens you is the machine that the baby will be kept in, as it alarms since you will be wondering what is happening, you see.’ (P5)

The lack of adequate orientation to the NICU environment seemed to increase the mothers’ anxiety and fear of coping:

‘We could have been told that the people in the neonatal unit are different from those outside the unit. Do not be surprised by what you will see, because they do not breathe on their own, they are assisted to breathe.’ (P7)

This participant expressed her concern about the effect on the mothers who observed infants who did not appear to be breathing and might even be dead:

‘Other things which were frightening are babies who were dying. You will find a baby who has long died kept for a long time. It is you who will tell yourself that the baby is dead … isn’t it that the baby has to breathe.’ (P7)

The initial loss of weight (not uncommon in preterm infants) was a source of great fear for the mothers:

‘…is weight dropped, dropped and dropped… I wondered if he would end up weighing 500 g as his weight dropped … I heard that some babies were weighing 650 g and these are the babies who were going [*dying*] … I also wondered if my baby is also going.’ (P7)

Participants began to realise that, despite the care, not all infants survived, and every visit to the unit created increased anxiety:

‘What is frightening is that we are sleeping with other mothers who also have babies in the same room as mine. Sometimes when we come, maybe coming for a three o’clock feed or any other time, you will find that baby dead. That is what frightened me thinking that maybe one day, when I come here, I will be told that my baby is no longer alive.’ (P8)

Cleanliness became an important concern for mothers, as this was deemed to be a measure of the quality of care: ’It’s … where the babies are, is very clean. It seems as if they clean every time we leave. I have never seen that place dirty.’ (P8)

## Relationships with health professionals

Positive interactions with the healthcare providers, particularly nurse-midwives, created a sense of partnership and facilitated communication. Participants felt reassured when the staff members answered their questions and provided adequate explanations:

‘There is good interaction this time. I am considering the fact that you will be asking the nurses about the condition of the baby, she will be patient enough to explain.’ (P8)

Although it was difficult to wait for assistance, participants appreciated that staff informed them when they could assist immediately: ‘There is good communication between the mothers and the nurses. It is only that sometimes it is very difficult to accept some situations as they come.’ (P8)

It was important for the participants to be clearly informed of procedures and tests and what these meant:

‘They did the chest X-ray and the head X-ray… Then they told me that the X-ray revealed bleeding from the lungs. The way they told me the disadvantages of bleeding from the lungs … I ended up accepting the situation .…’ (P3)

Being listened to was important, especially by the doctors, whom the mothers felt were more willing to listen than the nurses. Participants felt that the doctors listened to them and respected their concerns:

‘Sometimes when I tell them that my baby is on and off, they listen to me. Last time they wanted to discharge the baby but I told them that the baby has changed. I did not want the nurse to do that on my behalf and they listened to me.’ (P4)

The support from the health professionals was important to help them cope with caring for their tiny infants: ‘I was afraid of him … not knowing how I am going to handle him…. There is a nurse who told me not to be afraid of him because it is me who is going to take care of him while they show us how to take care of them’ (P1). For this participant, the birth of a preterm infant had been especially stressful, as she had lost a child born prematurely the previous year. This had reawakened her memories of the loss and raised fears that this infant too would die.

The support from the nurse had helped her to cope:

‘She then comforted me and told me to focus on the now and forget about the past. She said we should focus on the positive side and hope that the baby will be well. I felt better after talking to that nurse.’ (P8)

Sometimes negative staff interactions were frustrating for the participants, who ascribed these to lack of interest and personal problems of the nurses. Although they appreciated that the healthcare providers had numerous other responsibilities, they were concerned that, in busy times, routine care such as nappy changing was neglected:

‘What made me sad is … you will find your baby there … not taken care of … If you happen not to go to the unit because you were not feeling well, by the time you go there to check on her, you will find her in the same sheets and the nappy not changed.’ (P6)

Mothers wanted to be to feel included, informed of treatment changes and be involved in decision making and about their infant’s progress, especially when they were not with their infants:

‘All we do is to go, do what we are supposed to do and go back. They never tell us what has changed and what has not changed and how the baby is doing … sometimes you would find blood on the sheets, not knowing whether the baby was injected or what and they would not tell you.’ (P6)

Participants wanted to have more support and assistance. This mother explained how she felt when she reported that her infant’s feeding tube had been dislodged:

‘… the feeding tube that she used to drink with was out and when you tell the nurse that it is out. He/she would tell you to feed your baby with a cup. Sometimes when lifting the baby to change the sheets, he/she would say, “lift that baby, she is yours.”’ (P6)

A participant described her experience of an interaction with a busy doctor:

‘… when you come to the doctor and tell him that you found him doing this on the baby in order for him/her to explain what he/she was doing, he/she would tell you to go and ask the nurse.’ (P5)

Lack of emotional support or counselling by staff increased the mothers’ anxiety:

‘… I think I was not welcomed the first day. Even today there is no one who ever told me that you delivered a premature infant … because I thought I needed counselling, you see yet I told myself that I should be strong.’ (P7)

There was a feeling that they were not respected by the staff:

’…when a person talks to you like that yet you want your baby to get well, to grow and be discharged … you can end up being hurt if you are not able to control your feelings.’ (P8)

## From fear to emotional connection

Emotionally unprepared, it was initially very difficult for the mother to engage with the new infant:

‘I was wondering why it happened to me to give birth to such … to such a small baby, I became too emotional and left him.’ (P2)

A mother who had previously delivered full-term infants found her first encounter with her tiny infant especially stressful:

‘…I did not expect to deliver a preterm baby because it has never happened to me. It was such a shock to me … I cried while looking at him wondering whether … mmm … thinking that he is not a baby even if people can say what.’ (P7)

Over time, the participants overcame their fear as they were able to touch and hold their infants:

‘Initially, I used to go to the unit and when I arrived there, I used to just stand next to him, failing to touch him, wondering what I am going to do … now happy that I was able to hold the baby.’ (P1)

As the participants’ feelings became more positive towards their infants, their initial apprehensiveness abated:

‘My feelings have changed … I was afraid of him at first but I have now accepted him. I manage to lift him when I get there. Even when I am in the room I miss him. I have accepted him and I love him.’ (P2)

Participants became more confident as they touched and handled the infants and realised that, although tiny and preterm, their infants were just like any other infant. Touch and contact through feeding their infants made the connection with them real and tangible and facilitated the mother–infant relationships:

‘So when I started feeding him, I was able to touch him by then … Hee! I gave him a lot of love.’ (P3)

As the tiny infant began to respond, mothers responded in turn:

‘So it was very nice, I used to just look at him … even when I touched him before, I could feel that yes he is alive but there was no … But the day I saw him open the eyes I was not scared at all.’ (P7)

The fear and anxiety lessened, and mothers experienced a surge of love. This participant, whose first infant had died, was so afraid that her new preterm infant would not survive that she found it difficult to connect with him, explained how her feelings began to change:

‘I started loving him. At first I gave up because I had a baby who died.’ (P8)

## An enabling support network

Different support systems assisted the participants to cope with their difficulties while they were involved in the care of their preterm babies. With adequate information, staff support and encouragement, mothers were able to participate in their infants’ care and began to increase in confidence:

‘They also encourage us and tell us that if he is like this, you do this and that. They tell us that the baby will survive and it is possible.’ (P2)

Support, information and encouragement from other mothers was valued:

‘Some mothers who were there are the ones that comforted me by saying, please touch him, kiss him. I started touching his legs and toes’ (P7).

The participants supported and encouraged each other:

‘Like now, when one mother is sad, I am able to tell her that I was in the same situation, do not be sad. It will be fine … like there is a certain mother whose baby is in a coma … this is the fourth day, but I always tell her that it will be fine.’ (P7)

Visits and phone calls by family members reduced the participants’ isolation and enabled them to focus on the infant’s care. This participant’s mother appreciated her mother’s support:

‘My mother phoned me more often, telling me that I should not be scared because that my baby survives just like the child of … [*an acquaintance*]. He was born premature and here he is working for himself.’ (P7)

Religious beliefs were an important resource. Prayer provided a sense of comfort and hope:

‘God is great because even when I was in labour … I normally do not pray but after hearing that the baby is not alive, I talked to God and prayed seriously. I prayed and God answered my prayers’ (P3).

## Discussion

This study described the lived experience of mothers whose preterm infants were separated from them and nursed in the NICU. The mothers’ psychological preparation for a new infant, interrupted by sudden unexpected birth, affected their readiness for motherhood. Acceptance of the pregnancy and the physical and psychological changes that are associated with pregnancy is vital in order to deal with feelings of being a mother, as the attachment process commences during pregnancy (Alhusen, Hayat & Gross 2013). The mother’s emotional and physical experiences in relation to her unexpected and traumatic birth engenders fear for her infant’s safety, reduced confidence in interacting with her infant and may delay the attachment process (Fegran, Helseth & Fagermoen 2008; Korja *et al*. 2009).

The ability to communicate is needed by both the parent and child to build a relationship. Infants show attachment behaviours such as crying, smiling, grasping, reaching out and establishing visual contact to maintain proximity with their parents and communicate their needs. Preterm babies are less active and alert, less responsive to touch and less able to provide clear signals of distress (Korja *et al*. 2009); thus, the mother’s attachment process may be interrupted. Proximity through touch is reported to be the most powerful communication tool parents utilise in interacting with the infant (Fegran *et al*. 2008) and fear that their actions could harm the infant may inhibit contact (Fegran *et al*. 2008; Tilokskulchai *et al*. 2002). The anxiety that emanates from separation, lack of information, lack of explanation and fear for the survival of the preterm infant points to the intensity of the early bonding relationship between mother and child, her need to feel that she is the person who not only *has* the responsibility but *can* take responsibility for the care of her newborn. The threat of loss leads to anxiety; anxious mothers tend to experience higher levels of concern and increased vigilance (Rowe, Gardner & Gardner 2005), which in turn complicates attachment (Flacking *et al*. 2012; Holditch-Davis *et al*. 2009; Korja *et al*. 2009).

Despite the evidence (Franklin 2006; Shin & White-Traut 2007) that the NICU environment has the potential to overwhelm mothers and affect the attachment process, this study shows that mothers want to be part of their infants’ care. The balance between the need for mothers to feel that their infants have the best chance at survival and their need to feel a part of their infants’ care is difficult to achieve (Jackson, Ternestedt & Schollin 2003). The family is the context within which an intimate link between parents and infant is made, but the NICU environment creates a barrier, which could inhibit interaction and bonding. It is important, in the clinical context, to decrease maternal stress and promote close mother–infant contact (Korja, Latva & Lehtonen 2012).

Physical separation of mother and infant when the infant requires care in the NICU affects maternal–infant attachment and increases anxiety. Active parent involvement in the care of their preterm infant in the NICU is thought to moderate psychological stress and promote parent–infant attachment (Franck & Spencer 2003). Efforts to reduce anxiety, barriers to touch and skin contact should be made, as well as the provision of information that facilitates understanding and co-operation with the health professionals.

Effective and supportive communication from the health professionals in the NICU is vital for the mother’s well-being and the infant’s progress. Mothers need regular information about the infants’ progress and care (Wigert *et al*. 2006) but this in turn is challenging for nurses and doctors, whose focus is on the management of the vulnerable infant (Fegran *et al*. 2008). Nurses who are sensitive to the needs of mothers are helpful in guiding and strengthening maternal responses to their infants, thus promoting attachment (Kearvell & Grant 2010). A social interaction or ‘chat’ strategy, in which nurse and mother connect, not only in relation to the infant’s care but also about life outside the nursery, has been found to be beneficial in developing rapport (Aagaard & Hall 2008; Fenwick, Barclay & Schmied 2001). An organisational culture which supports the formation of parent–infant relationships is recommended by Flacking *et al*. (2012) to empower parents and include them as partners in care.

When mothers were provided with information on how to care and were also shown how to provide the caring activities, they developed confidence in taking care of their preterm baby. Aagaard and Hall (2008) suggest that facilitative actions, which include giving empowering and consistent information, are important in helping mothers to develop care-giving actions for the infant, which in turn facilitate attachment. These actions include ensuring that mothers are well informed, the development of good parent–staff relationships, open visiting to infant, breastfeeding support, promotion of physical parent–infant contact and regular updates of the infant’s progress. Appropriate, timely and sensitive provision of information is a fundamental need for parents. Regular communication with parents of preterm infants allows them to be partners in care giving and decision making, and empowers the mother to fulfil her role, increases self-confidence and engenders a sense of control and feeling of being connected to her infant (Griffin 2006). Despite the overwhelming nature of such information, parents find it more stressful when information is withheld.

In this study, the relative absence of fathers is not unusual, given the cultural context, wherein fathers do not play an active role in the initial care of a newborn infant. In other studies, however, the importance of the involvement of the father has been stressed (Fegran *et al*. 2008; Lindberg & Öhrling 2008).

Touch, one of our basic needs in communication, is one of the most fear-inducing responses in the connection between mothers and their preterm infants. With the encouragement of the nurses, they were able to overcome their fear and were able to hold their preterm infant with confidence. The difficulties that mothers of preterm infants face before they are able to initiate contact with their infant can delay the building of a relationship between mother and baby.

Taking care of a fragile infant in an unfamiliar environment was distressing for the participants in our study, and the staff support through provision of information, encouragement and demonstration facilitated the attachment between participants and their preterm infants Support that parents receive from staff is helpful in developing competence and attachment relationships with the baby (Mok & Leung 2006; Schenk, Kelley & Schenk 2005). Mothers who receive reassurance regarding their infant’s conditions were able to interact with their infants in a positive manner throughout their hospitalisation periods (Mok & Leung 2006). Support from other mothers was also helpful, particularly the encouragement from mothers whose infants had been in the NICU for a few days, could be considered ‘experienced’ and could provide information and encouragement to the mothers whose infants had more recently been admitted to the NICU.

## Limitations of the study

This limited study explored the lived experience of eight mothers whose preterm babies were admitted to one NICU in Botswana. The interviews were conducted in Setswana and translated into English, which might have influenced the interpretation of the lived experiences. In the Setswana culture, the father has a limited role in the early days of a newborn’s life; it is necessary that the experiences and role of the father with regard to a preterm infant should be understood. As a small qualitative study, the findings are not generalisable, although may be transferable to similar settings.

## Recommendations

The health of the mother, preterm infant and family is a critical aspect of maternal and child health care. Women should be encouraged to learn as much as possible about the birth process in order to raise awareness of the possibility of preterm birth and how this is managed in maternal care settings. Information about the possibility of preterm labour and delivery is seldom provided to pregnant women. The first-time mothers in this study had never seen a preterm infant and had no previous exposure to an incubator or the NICU. Although a number of topics are covered in the form of brochures for mothers, information on preterm birth and care of a preterm infant is not freely available. Pregnant women need to be sensitively informed about the possible complications of pregnancy, preterm delivery and the available interventions that are provided, even though most infants would not need specialised care (Herbst & Maree 2006).

Mothers should have open access to the neonatal unit so that separation is minimised, mothers’ stress is minimised and mother–infant interaction is enhanced (Flacking *et al*. 2012; Franklin 2006). Each neonatal unit should have an orientation package for parents, stipulating what they are expected to do and informing them what they should expect while in the NICU (Herbst & Maree 2006). Mothers of newly admitted infants should be orientated to the unit and counselled regarding the infant’s condition and the treatment options. Support groups are a useful resource to assist the new mothers in learning from others with similar experiences. Fathers should be involved as much as possible, although infection control protocols, may limit this involvement.

Mothers would also benefit from the provision of a comfortable space as close as possible to the NICU in which they could relax, interact with other mothers in similar situations or be supported by family members. Counselling, health literacy skills and promotion of kangaroo mother care should be included in all health professional curricula.

## Conclusion

The findings of this study demonstrate that health professionals in neonatal settings have a dual responsibility – to the infant and to the mother. Through the supportive promotion of mothers’ participation in the care of their preterm infants, the mother–infant emotional connection is facilitated, which is vital for the survival of the infant and the well-being of the mother.
